# Genotype-phenotype correlation of ocular von Hippel-Lindau disease in Koreans

**DOI:** 10.1371/journal.pone.0311665

**Published:** 2024-10-07

**Authors:** Sungsoon Hwang, Se Woong Kang, Jong-Won Kim, Sang Jin Kim

**Affiliations:** 1 Department of Ophthalmology, Samsung Medical Center, Sungkyunkwan University School of Medicine, Seoul, Republic of Korea; 2 Department of Laboratory Medicine and Genetics, Samsung Medical Center, Sungkyunkwan University School of Medicine, Seoul, Republic of Korea; 3 Department of Health Science and Technology, Samsung Advanced Institute for Health Sciences and Technology (SAIHST), Sungkyunkwan University, Seoul, Republic of Korea; IHRC, Inc. (Human Resource Service Administration), UNITED STATES OF AMERICA

## Abstract

This scientific report aims to investigate the genotype-phenotype correlations of retinal hemangioblastoma (RH) in von Hippel-Lindau (VHL) disease. The study included 77 patients with genetically confirmed VHL disease who visited an ophthalmology clinic for the evaluation of RH. The presence, location, and size of RH were evaluated, Patients were categorized into three groups based on variants: HIF-1α binding site missense (HM), non-HIF-1α binding site missense (nHM), and truncating (TR) mutations. Fifty-six patients (72.7%) had RH in either eye, and 24 had bilateral RH. Sixteen patients (20.8%) had juxtapapillary RH in either eye. Nine patients had RH ≥ 2.0 disc diameters in size. *VHL* c.208G>A variant was the most frequent single mutation. Compared with patients having nHM mutations (15 patients) in VHL gene, patients with HM mutations (33 patients) or TR mutations (26 patients) presented a greater number of eyes affected (p = 0.007 and 0.004, respectively), a greater number of RH (p = 0.012 and 0.003, respectively), and more frequent presentation of large RH ≥ 2.0 disc diameters (p = 0.012, and 0.013, respectively). In conclusion, this study provides a deeper understanding of the genetic spectrum of VHL disease in Korean VHL disease and highlights the importance of the location of missense mutations regarding the risk of RH.

## Introduction

Von Hippel-Lindau (VHL) disease is a genetic disorder characterized by tumor development in the retina, central nervous system, kidneys, adrenal glands, and pancreas [1, 2]. VHL disease is caused by pathogenic variants in *VHL* gene, a tumor suppressor gene located on the short arm of chromosome 3. The disease is a relatively common inherited cancer syndrome that occurs in 1 in 36,000 individuals worldwide [3, 4]. VHL disease is inherited in an autosomal dominant pattern; however, 20% of patients with VHL disease have de novo mutation at their generation [5].

The most common tumor that occurs in patients with VHL disease is hemangioblastoma, a benign blood vessel tumor in retina and central nervous system [2]. The prevalence of hemangioblastoma in patients with VHL disease varies depending on the study and population analyzed. However, it is generally estimated to be approximately 50–80% [2]. Ophthalmologists are mainly concerned with retinal hemangioblastomas (RH), as these tumors can frequently result in hard exudates, retinal edema, and tractional or exudative retinal detachment, which can cause vision loss [6, 7]. Regular surveillance of VHL disease with an ophthalmologist is crucial, as it can pose challenges in treatment with the later detection of RH and there is always a risk of recurrence and new tumor formation [8].

Advances in genetic sequencing technology have made genetic testing more efficient and lowered costs. With the increasing accessibility of genetic testing, there is a growing interest in understanding the genotype-phenotype correlation of diseases. Regarding RH in VHL disease, although a few studies have examined the relationship between genotype and phenotype, the results of these studies have been inconsistent [9–13]. Moreover, most previous studies did not include Asian population and there is a possibility that these associations may vary among different ethnic groups. This highlights the need for further research, particularly studies that include Asian population to gain a deeper understanding of the genotype-phenotype correlation of RH in patients with VHL disease.

Therefore, the present study aimed to analyze the genotype profile of Korean patients with VHL disease, and examine the relationship between the genetic makeup and observable traits of RH.

## Materials and methods

### Study design and ethical approval

This retrospective study included consecutive patients with genetically verified VHL disease who underwent ocular surveillance for RH. The study adhered to the tenets of the Declaration of Helsinki and was approved by the Institutional Review Board of Samsung Medical Center, Seoul, Republic of Korea (IRB number 2022-05-023). The board waived the requirement for informed consent based on the retrospective nature of the study.

### Subjects

**Fig 1:**
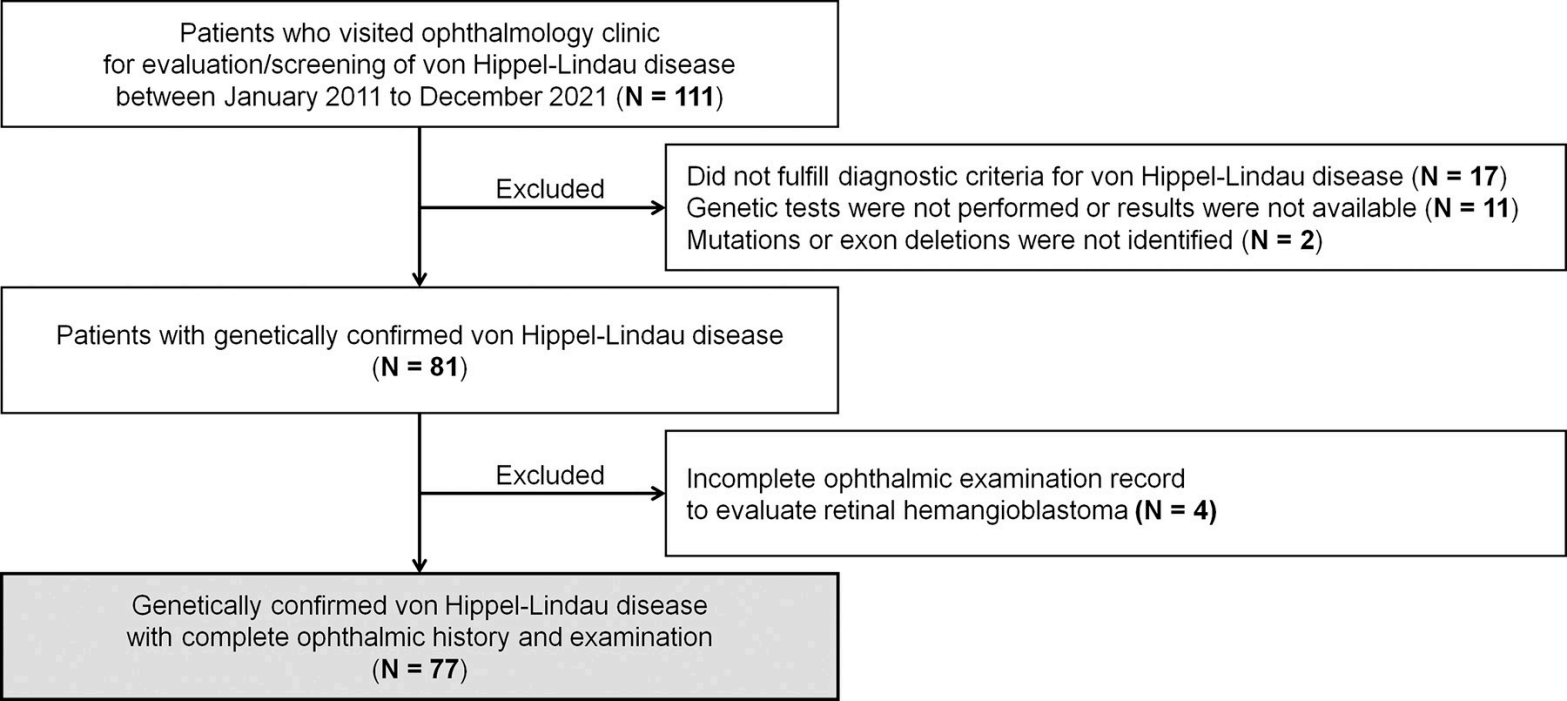
Flow chart of the selection of patients with von Hippel-Lindau disease.

The medical records of all consecutive patients screened for VHL disease between January 2011 and December 2021 at the Samsung Medical Center, Department of Ophthalmology (by SH, SWK, and SJK) were retrospectively reviewed. A diagnosis of genetically verified VHL disease was established for patients with pathogenic or likely pathogenic variant of the *VHL* gene according to the guidelines of the American College of Medical Genetics and Genomics (ACMG) [14] or who had a large deletion in any exon of the VHL gene. Individuals were excluded from the study if their ophthalmic examination records were incomplete and could not be used to evaluate the presence, number, location, and size of RH (Fig 1).

### Clinical protocol for evaluation of ocular VHL disease

The Samsung Medical Center has adopted a multidisciplinary approach to managing VHL disease. In 2011, the center established the VHL clinic, which is staffed by specialists, including endocrinologists, urologists, general surgeons, neurosurgeons, ophthalmologists, otolaryngologists, radiologists, and clinical pathologists [15]. The clinic offers surveillance and tailored genetic counseling, and patients are promptly referred to the relevant department for early detection of clinical manifestations associated with VHL disease. Detailed information on this multidisciplinary approach is available elsewhere [15].

As part of a systematic multidisciplinary approach to VHL disease, patients underwent a comprehensive ocular examination to screen and evaluate the ocular involvement of VHL disease. The examinations included measurement of best-corrected visual acuity (BCVA), manifest refraction, applanation tonometry, slit-lamp biomicroscopy, and dilated fundus examination. Additionally, ultrawide-field fundus photography and fluorescein angiography (uWF-FP and uWF-FA, Optomap; Optos Plc, Dunfermline, Scotland) were routinely conducted. Treatment and follow-up plans were then determined and implemented at the discretion of the treating clinician.

### Genetic testing and variant analysis

Genetic mutations associated with VHL disease were identified using the following protocol: blood samples were collected from patients, and genomic DNA was extracted from peripheral blood leukocytes using the Wizard Genomic DNA Purification Kit (Promega, Madison, WI, USA). The exonic and flanking intragenic regions of all three exons of the VHL gene were sequenced using an ABI 3130XL Genetic Analyzer (Applied Biosystems, Foster City, CA, USA) and a BigDye Terminator v3.1 Cycle Sequencing Kit (Applied Biosystems). In instances where pathogenic variants were not identified through direct sequencing, multiplex ligation-dependent probe amplification (MLPA) analysis (SALSA MLPA Probemix P016 VHL; MRC Holland, Amsterdam, Netherlands) was performed to detect large genome rearrangements. The results of the MLPA analysis were analyzed using the GeneMarker software (SoftGenetics, State College, PA, USA).

### Data collection

The investigators collected clinical data during the initial ophthalmic evaluation at the Samsung Medical Center. The data included demographic details, systemic organ involvement in VHL disease, BCVA on the Snellen scale, the number, location, and size of RH, and the presence of RH-related complications. Ocular involvement was categorized as no, unilateral, or bilateral involvement. The total number of tumors in both eyes of each individual was categorized as 0, 1, 2–4, and ≥5, and the presence of juxtapapillary tumors was recorded. The size of the largest tumor involved was measured and categorized into three groups based on the measurement of the longest diameter: less than 1.0 disc diameter (DD), between 1.0 DD and less than 2.0 DD, and 2.0 DD or greater. Ocular complications were noted, including preretinal membranes, macular exudates, and tractional/exudative retinal detachment. VHL variants were classified as missense, nonsense, splicing, frameshift, in-frame deletions, or exon deletions.

### Statistical analysis

A descriptive analysis examined the baseline demographics and initial clinical and ocular characteristics of patients with genetically confirmed VHL disease. To evaluate the relationship between ocular phenotype and genotype, variants were grouped into three categories: truncating (TR) mutations (exon deletions, frameshift, nonsense, and splicing mutations) and missense mutations, which were further subdivided into hypoxia-inducible factor-1 alpha (HIF-1α) binding site missense (HM, residues 65–117) and non-HIF-1α binding site missense (nHM) mutations. Ocular involvement, number of tumors, size of the largest tumor, and juxtapapillary involvement were compared using the Mann–Whitney U for ordinal variables and Fisher’s exact tests for binary variables. P-values were two-sided and considered statistically significant if they were less than 0.017, accounting for multiple comparisons among the three genotype groups using the Bonferroni method. All statistical analyses were performed using SPSS software version 25.0 (IBM, Armonk, NY, USA).

## Results

Among the 111 patients who visited the ophthalmology clinic for evaluation/screening of VHL disease between January 2011 and December 2021, 77 patients with a genetic diagnosis of VHL disease and a complete ophthalmic history and examination were included in the study (Fig 1).

Table 1 describes the detailed baseline characteristics of the participants. The average age was 34.9 years old, and 35 patients (45.5%) were male. Among these, 56 patients (72.7%) had RH in either eye. Table 2 demonstrates the ocular characteristics of patients with VHL disease. Bilateral involvement of RH was observed in 24 patients (31.2%), 10 patients (13.0%) had 5 or more tumors, 16 patients (20.8%) had juxtapapillary tumors, and 9 patients (11.7%) had tumors greater than 2 DD in either eye.

**Table 1:** Baseline characteristics of patients with genetically verified von Hippel-Lindau disease.

Characteristics	Values
Number of patients, No.	77
Number of unrelated families, No.	62
Age, years, mean ± SD	34.9 ± 13.3
Sex, No. (%)	
• Male	35 (45.5)
• Female	42 (54.5)
**Ocular involvement and evaluation**	
• Number of patients with RH, No. (%)	56 (72.7)
• Age at initial diagnosis of RH, mean ± SD	34.1 ± 13.9
• Prior ablative treatment history for RH, No. (%)	10 (13.0)
• Fundus photograph images available, No. (%)	77 (100.0)
• Ultrawide-field fundus photograph images available, No. (%)	71 (92.2)
• Ultrawide-field fluorescein angiography images available, No. (%)	65 (84.4)
**Systemic organ involvement, No. (%)**	
• Central nervous system involvement	
• Cerebrospinal hemangioblastoma	62 (80.5)
• Endolymphatic sac tumor	3 (3.9)
• Visceral organ involvement	
• Renal cell cancer/renal cyst	44 (57.1)
• Pancreatic neuroendocrine tumors/pancreatic cyst	35 (45.5)
• Pheochromocytoma/paraganglioma	22 (28.6)
• Epididymal/uterine ligament cystadenoma	4 (5.2)

SD, standard deviation; RH, retinal hemangioblastoma

**Table 2:** Characteristics of retinal hemangioblastoma in patients with genetically verified von Hippel-Lindau disease.

Characteristics	Values
**Number of patients with tumors, No. (%)**	56 (72.7)
**Number of eyes involved, No. (%)**	
• No involvement	21 (27.3)
• Unilateral involvement	32 (41.6)
• Bilateral involvement	24 (31.2)
**Number of tumors in each individual, No. (%)**	
• 0	21 (27.3)
• 1	20 (26.0)
• 2–4	25 (32.5)
• ≥ 5	10 (13.0)
• Unable to categorize to evisceration/phthisis	1 (1.3)
**Location, No. (%)**	
• Juxtapapillary tumor in either eye	16 (20.8)
**Size, No. (%)**	
• ≥ 1.0 disc diameter in either eye	24 (31.2)
• ≥ 2.0 disc diameter in either eye	9 (11.7)
**Ocular complications, No. (%)**	
• Preretinal membrane in either eye	13 (16.9)
• Macula exudates in either eye	10 (13.0)
• Tractional/exudative retinal detachment in either eye	9 (11.7)
• Papilledema d/t increased intracranial pressure	2 (2.6)
**Visual impairment, No. (%)**	
• < 20/40 in either eye	14 (17.3)
• < 20/400 (legal blindness) in either eye	7 (9.3)

**Fig 2:**
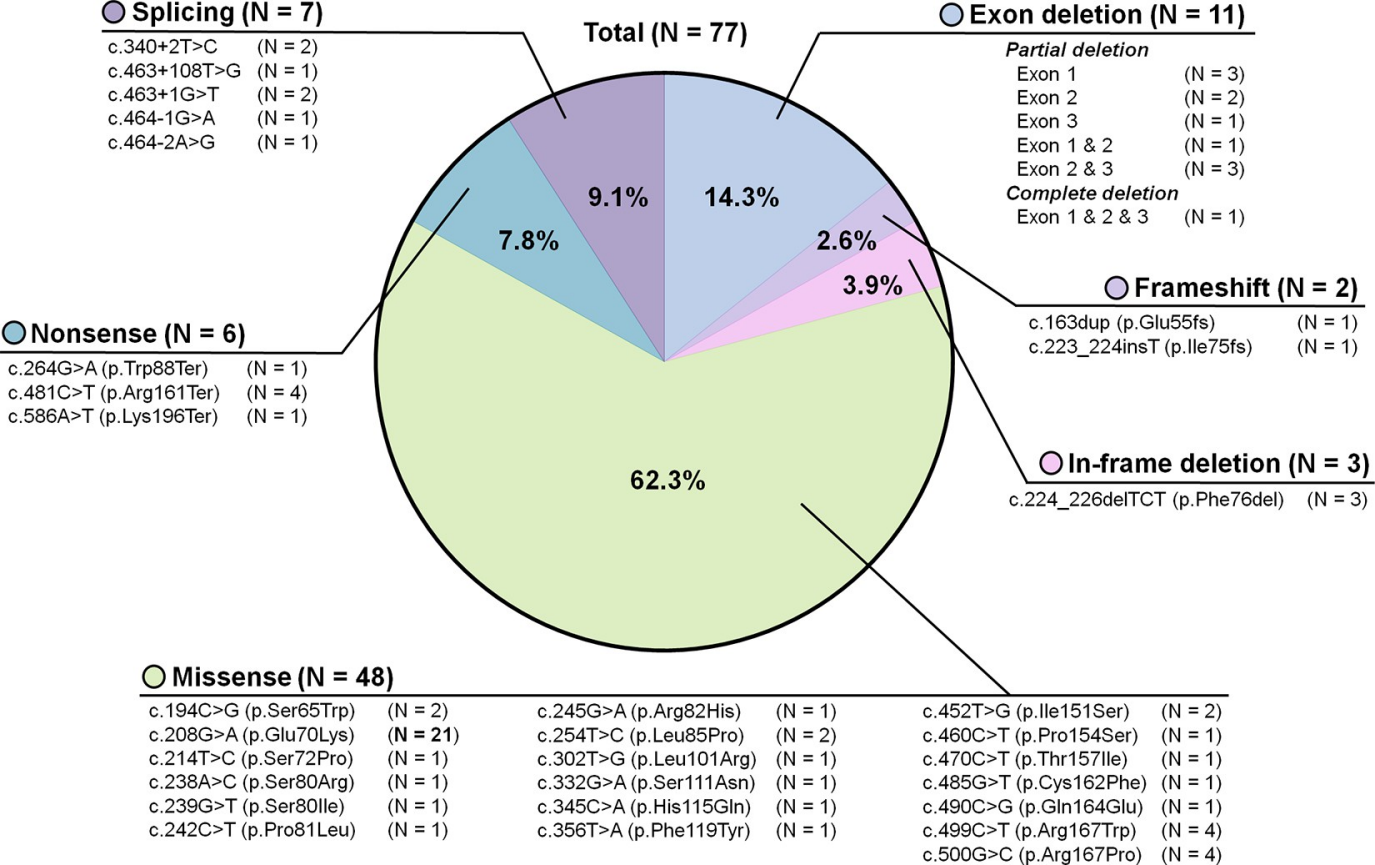
Mutation profile of patients with von Hippel-Lindau disease in Korea.

Fig 2 illustrates the variant profile of the VHL gene in Korean patients with VHL disease. Of the 77 patients included in the study, 26 (33.8%) had TR mutations, and 48 (62.3%) had missense mutations, of which 33 (42.9%) had mutations at the HIF-1α binding site (HM group), and 15 (19.5%) had mutations outside the HIF-1α binding site (nHM group). The c.208G>A variant was the most common mutation observed in the study participants.

**Fig 3:**
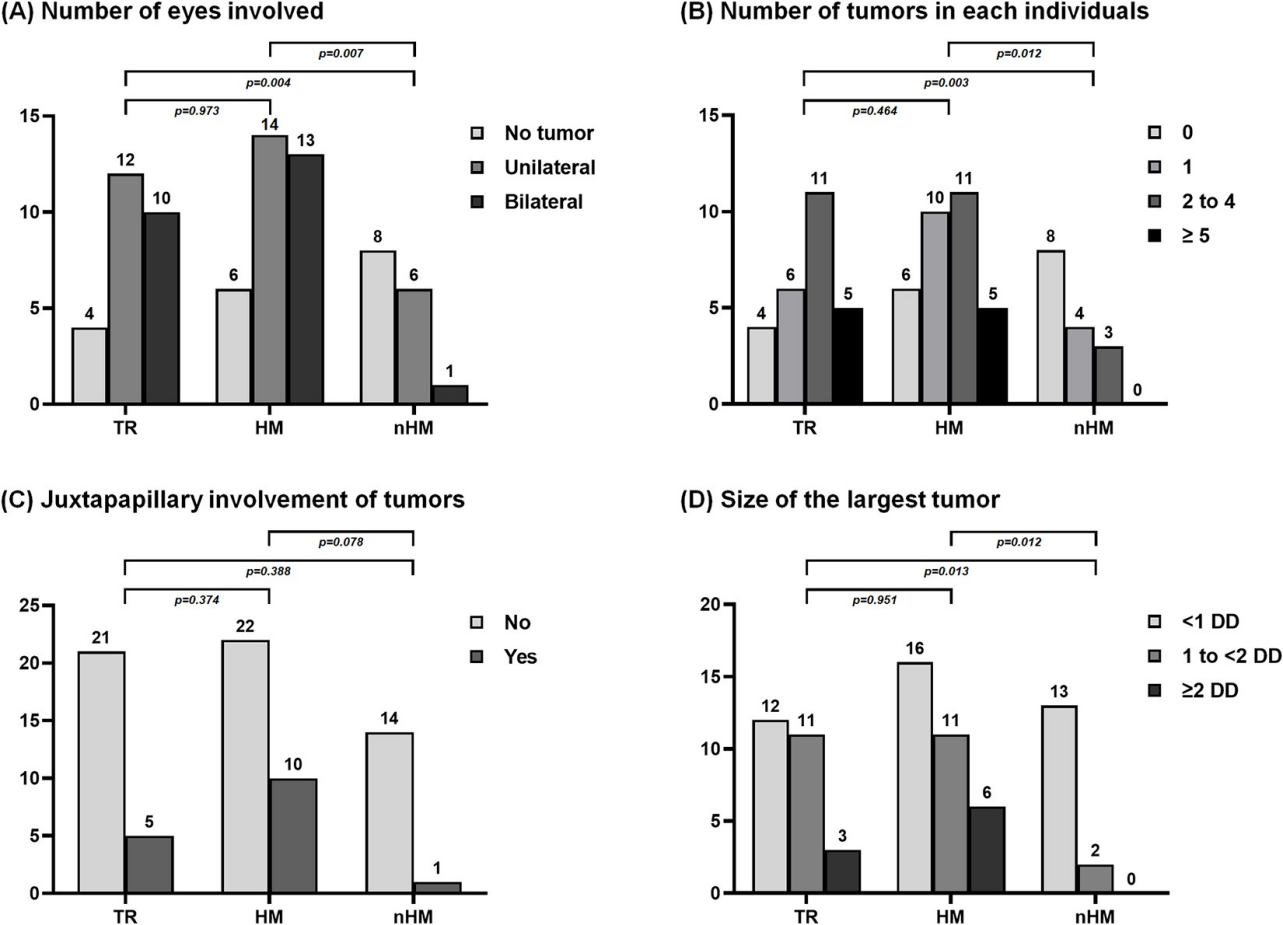
Genotype-phenotype correlation of retinal hemangioblastoma in patients with von Hippel-Lindau disease. The phenotypes of retinal hemangioblastoma were compared among three groups of mutations: (1) the truncating variant group (TR) which included exon deletion, nonsense mutation, and frameshift mutation; (2) the hypoxia-inducible factor-1alpha binding site missense mutations group (HM); and (3) the non-hypoxia-inducible factor-1alpha binding site missense mutations group (nHM). The Mann-Whitney test shows that individuals in the TR and HM groups had greater number of eyes involved, a greater number of tumors overall, and a larger tumor size than those in the nHM group.

Fig 3 illustrates the correlation between the genotype and phenotype of RH in patients with VHL disease. Of the 15 patients with nHM mutations, eight (53.3%) had no tumors. In contrast, only 4 (15.4%) of the 26 patients in the TR group and 6 (18.2%) of the 33 patients in the HM group were tumor-free (Fig 3A and 3B). Additionally, none of the patients in the nHM group had more than three tumors (Fig 3B), and all tumors in this group were less than 2 DD in diameter (Fig 3D). The statistical analyses showed that compared to the nHM group, the TR and HM groups had a higher number of eyes affected by RH (p = 0.004 and 0.007, respectively), a greater number of overall tumors (p = 0.003 and 0.012, respectively), and a larger size of the largest tumor (p = 0.013 and 0.012, respectively). No statistically significant differences were found among the groups regarding juxtapapillary involvement of RH, presence of premacular membrane, macular hard exudates, exudative/tractional retinal detachment, visual acuity, or age. Representative fundus photographs and fluorescein angiography images for each group (TR, HM, and nHM) are illustrated in S1 Fig.

## Discussion

This study revealed that *VHL* c208G>A variant is highly prevalent in Korean patients with VHL disease. TR and HM variants have a greater risk of RH than nHM variants in VHL disease. The findings of this study emphasize the significance of the location of missense mutations, which has been overlooked in previous studies investigating the correlation between genotype and ocular phenotype in VHL disease.

Understanding the genotype-phenotype correlation in VHL disease requires a thorough understanding of the underlying pathomechanisms. The VHL protein plays a crucial role in various cellular processes, with its most important canonical pathway being the hypoxia-dependent pathway [16, 17]. In oxygenated conditions, a normally functioning VHL protein is responsible for the degradation of HIF-1α, a transcription factor that regulates the expression of genes involved in angiogenesis and cell proliferation, such as vascular endothelial growth factor (VEGF), platelet-derived growth factor (PDGF), erythropoietin (EPO), and transforming growth factor (TGF) [18, 19]. However, under hypoxic conditions, the binding of HIF-1α to VHL protein is hindered, resulting in the upregulation of the aforementioned genes [18]. Individuals with VHL disease possess pathogenic variants in one allele of the two VHL alleles. If a cell harbors additional mutations in another normal VHL allele, the normally functioning VHL protein is lost. As a consequence, HIF-1α is no longer properly degraded, even under oxygenated conditions, leading to a pseudohypoxic condition and activated expression of VEGF, PDGF and EPO genes, resulting in the formation of tumors, such as hemangioblastomas and renal cell carcinomas [20].

Previous studies investigating the genotype-phenotype correlation of ocular VHL disease have yielded conflicting results [9–13]. The majority of these studies have compared the ocular phenotype between missense mutations and TR variants, with some reports finding no significant differences between the two genotype categories with regard to RH, while others have suggested that either one category may be associated with better or worse outcomes. For example, Wong et al. reported no difference in the unilaterality or bilaterality of ocular disease or the number and extent of tumors between missense mutations and TR mutations [10]. In contrast, Ong et al. suggested that TR variants are associated with a higher risk of retinal angioma than in missense or single deletion mutation [9] and Reich et al. reported that individuals with TR variants developed RH earlier and had a higher number of RH and greater risk of enucleation/phthisis [13]. On the other hand, Binderup et al. reported that individuals with missense mutations had a greater risk of incident RH compared to those with TR mutations [11] and Hajjaj et al. reported that missense mutations in patients with VHL disease had a higher prevalence of progression-related complications [12]. The conflicting results in past ophthalmic studies may be because the heterogeneous phenotype of each missense mutation was grouped into a single category, the varying distribution of missense mutations depends on the study population, and ethnicity-specific genetic backgrounds can significantly influence disease manifestations.

Unlike TR mutations that produce a shortened version of an often deleterious protein, missense mutations result in a single amino acid substitution in a protein. Recent research has demonstrated that missense mutations can have varying phenotypes depending on their location within the structural domains of the VHL protein [21–23]. A previous bioinformatic study has indicated that impairment of HIF regulation is significantly associated with the occurrence of hemangioblastoma [24]. Proper attachment of VHL to HIF-1α is the first step of VHL-mediated degradation of HIF-1α, with the attachment site being located within residues 65–117 [25, 26]. Hence, mutations within this region have been suggested to greatly reduce the ability of VHL to degrade HIF-1α, which is likely to be associated with a worse phenotype of VHL disease [21]. Genotype–phenotype studies that differentiate missense mutations by location have reported that HM variants are associated with a higher risk of central nervous system hemangioblastoma and poorer overall survival than in nHM variants [22, 23]. This study subdivided missense mutations based on their location within the structural domain, as the degree of impairment of HIF regulation may differ depending on the location of the missense mutation. As hypothesized, our results suggest that HM variants have a higher risk of RH than in nHM variants and that the ocular phenotype of HM variants was comparable to that of TR variants.

The c.208G>A missense variant was highly prevalent in the study population of this study. Previous studies on VHL disease have reported that this mutational hotspot is prevalent in the Korean population and is not commonly observed in Western populations or even in other East Asian populations, such as China or Japan [27]. The c.208G>A missense mutation is an HM variant based on its location and is functionally predicted to cause impairment in HIF-1α binding [27, 28]. Given that c.208G>A has been previously reported to be associated with multiple CNS hemangioblastomas and RH [27, 29], the results of this study support the notion that the HM variants carry a high risk of RH.

Currently, VHL surveillance protocols are standardized regardless of genotype. However, considering our findings that HM and TR mutations are associated with a higher risk of RH, along with other studies indicating that the risk of central nervous system hemangioblastoma, renal cell carcinoma, and pheochromocytoma can vary by genotype [21–23], implementing targeted screening based on genotype in VHL patients could be beneficial. Additionally, genetic counseling and family planning could also be individualized based on genotype. Establishing genotype-specific surveillance protocols would require robust evidence of genotype-phenotype correlations and a thorough evaluation of cost-effectiveness. Our study results may lay the groundwork for such targeted screening, but further research is necessary to solidify these genotype-phenotype correlations and assess the practical implications.

The strength of this study is that it established a meaningful genotype–ocular phenotype correlation in patients with VHL disease by carefully grouping genetic variants and conducting a thorough analysis of RH-related phenotypes. However, this study has certain limitations that need to be addressed. First, the sample size was insufficient for a more detailed genotype-phenotype grouping. Second, the study only used data from the initial evaluation and did not include longitudinal data; therefore, it is impossible to know how clinical presentations progressed or how patients responded to treatment based on their genotype. To address these limitations, future studies that include larger multi-center patient samples and longitudinal data are necessary for precise understanding of the risk of RH and progression in different genotypes.

In conclusion, this study revealed that TR and HM mutations predispose patients with VHL disease to a greater risk of RH than in other missense mutations in patients with VHL disease. The findings of our study will provide insights for genetic counseling and management of patients with VHL disease. We suggest that patients with TR and HM variants undergo aggressive ophthalmic screening for early detection and timely management of RH.

## Supporting information

S1 Fig Representative fundus photographs and angiographs for each VHL mutation group: (A) Truncating Variant Group (TR), (B) Hypoxia-Inducible Factor-1alpha Binding Site Missense Mutations Group (HM), and (C) Non-Hypoxia-Inducible Factor-1alpha Binding Site Missense Mutations Group (nHM). (A) A 45-year-old female with a heterozygous exon 2–3 deletion in the VHL gene, showing a large retinal hemangioblastoma with extensive exudation in the temporal periphery of the left eye. (B) A 46-year-old male with a heterozygous c.208G>A variant in the VHL gene, who is blind in the left eye due to retinal hemangioblastomas and has multiple retinal hemangioblastomas and traction membrane in the right eye. (C) A 55-year-old male with a heterozygous c.499C>T variant in the VHL gene, showing no retinal hemangioblastomas.
(DOCX)

## References

[1] Maher ER, Neumann HP, Richard S. von Hippel-Lindau disease: a clinical and scientific review. Eur J Hum Genet. 2011; 19: 617–623. doi: 10.1038/ejhg.2010.175 21386872 PMC3110036

[2] Lonser RR, Glenn GM, Walther M, Chew EY, Libutti SK, Linehan WM, et al. von Hippel-Lindau disease. Lancet. 2003; 361: 2059–2067. doi: 10.1016/S0140-6736(03)13643-4 12814730

[3] Maher ER, Yates JR, Harries R, Benjamin C, Harris R, Moore AT, et al. Clinical features and natural history of von Hippel-Lindau disease. Q J Med. 1990; 77: 1151–1163. doi: 10.1093/qjmed/77.2.1151 2274658

[4] Maher ER, Kaelin WG, Jr. von Hippel-Lindau disease. Medicine (Baltimore). 1997; 76: 381–391. doi: 10.1097/00005792-199711000-00001 9413424

[5] Richards FM, Payne SJ, Zbar B, Affara NA, Ferguson-Smith MA, Maher ER. Molecular analysis of de novo germline mutations in the von Hippel-Lindau disease gene. Hum Mol Genet. 1995; 4: 2139–2143. doi: 10.1093/hmg/4.11.2139 8589692

[6] Chew EY. Ocular manifestations of von Hippel-Lindau disease: clinical and genetic investigations. Trans Am Ophthalmol Soc. 2005; 103: 495–511. 17057815 PMC1447586

[7] Dollfus H, Massin P, Taupin P, Nemeth C, Amara S, Giraud S, et al. Retinal hemangioblastoma in von Hippel-Lindau disease: a clinical and molecular study. Invest Ophthalmol Vis Sci. 2002; 43: 3067–3074. 12202531

[8] Louise MBM, Smerdel M, Borgwadt L, Beck Nielsen SS, Madsen MG, Møller HU, et al. von Hippel-Lindau disease: Updated guideline for diagnosis and surveillance. Eur J Med Genet. 2022; 65: 104538. doi: 10.1016/j.ejmg.2022.104538 35709961

[9] Ong KR, Woodward ER, Killick P, Lim C, Macdonald F, Maher ER. Genotype-phenotype correlations in von Hippel-Lindau disease. Hum Mutat. 2007; 28: 143–149. doi: 10.1002/humu.20385 17024664

[10] Wong WT, Agrón E, Coleman HR, Reed GF, Csaky K, Peterson J, et al. Genotype-phenotype correlation in von Hippel-Lindau disease with retinal angiomatosis. Arch Ophthalmol. 2007; 125: 239–245. doi: 10.1001/archopht.125.2.239 17296901 PMC3019103

[11] Binderup ML, Budtz-Jørgensen E, Bisgaard ML. Risk of new tumors in von Hippel-Lindau patients depends on age and genotype. Genet Med. 2016; 18: 89–97. doi: 10.1038/gim.2015.44 25834951

[12] Hajjaj A, van Overdam KA, Oldenburg RA, Koopmans AE, van den Ouweland AMW, de Klein A, et al. Retinal haemangioblastomas in von Hippel-Lindau germline mutation carriers: progression, complications and treatment outcome. Acta Ophthalmol. 2020; 98: 464–471. doi: 10.1111/aos.14360 32003155 PMC7496349

[13] Reich M, Jaegle S, Neumann-Haefelin E, Klingler JH, Evers C, Daniel M, et al. Genotype-phenotype correlation in von Hippel-Lindau disease. Acta Ophthalmol. 2021; 99: e1492–e1500. doi: 10.1111/aos.14843 33720516

[14] Richards S, Aziz N, Bale S, Bick D, Das S, Gastier-Foster J, et al. Standards and guidelines for the interpretation of sequence variants: a joint consensus recommendation of the American College of Medical Genetics and Genomics and the Association for Molecular Pathology. Genet Med. 2015; 17: 405–424. doi: 10.1038/gim.2015.30 25741868 PMC4544753

[15] Yoon SJ, Kwon WK, Hong G, Jang JH, Jeong BC, Kim JH, et al. Genetic Counseling and Long-Term Surveillance Using a Multidisciplinary Approach in von Hippel-Lindau Disease. Ann Lab Med. 2022; 42: 352–357. doi: 10.3343/alm.2022.42.3.352 34907105 PMC8677470

[16] Hudler P, Urbancic M. The Role of VHL in the Development of von Hippel-Lindau Disease and Erythrocytosis. Genes (Basel). 2022; 13 doi: 10.3390/genes13020362 35205407 PMC8871608

[17] Hsu T. Complex cellular functions of the von Hippel-Lindau tumor suppressor gene: insights from model organisms. Oncogene. 2012; 31: 2247–2257. doi: 10.1038/onc.2011.442 21996733 PMC3343179

[18] Liao C, Zhang Q. Understanding the Oxygen-Sensing Pathway and Its Therapeutic Implications in Diseases. Am J Pathol. 2020; 190: 1584–1595. doi: 10.1016/j.ajpath.2020.04.003 32339495 PMC7416076

[19] Maina EN, Morris MR, Zatyka M, Raval RR, Banks RE, Richards FM, et al. Identification of novel VHL target genes and relationship to hypoxic response pathways. Oncogene. 2005; 24: 4549–4558. doi: 10.1038/sj.onc.1208649 15824735

[20] Kaelin WG Jr., The von Hippel-Lindau tumour suppressor protein: O2 sensing and cancer. Nat Rev Cancer. 2008; 8: 865–873. doi: 10.1038/nrc2502 18923434

[21] Qiu J, Zhang K, Ma K, Zhou J, Gong Y, Cai L, et al. The Genotype-Phenotype Association of Von Hipple Lindau Disease Based on Mutation Locations: A Retrospective Study of 577 Cases in a Chinese Population. Front Genet. 2020; 11: 532588. doi: 10.3389/fgene.2020.532588 33362845 PMC7762453

[22] Liu SJ, Wang JY, Peng SH, Li T, Ning XH, Hong BA, et al. Genotype and phenotype correlation in von Hippel-Lindau disease based on alteration of the HIF-α binding site in VHL protein. Genet Med. 2018; 20: 1266–1273. doi: 10.1038/gim.2017.261 29595810

[23] Xie H, Ma K, Zhang J, Hong B, Zhou J, Li L, et al. Novel genetic characterisation and phenotype correlation in von Hippel-Lindau (VHL) disease based on the Elongin C binding site: a large retrospective study. J Med Genet. 2020; 57: 744–751. doi: 10.1136/jmedgenet-2019-106336 32303605

[24] Forman JR, Worth CL, Bickerton GR, Eisen TG, Blundell TL. Structural bioinformatics mutation analysis reveals genotype-phenotype correlations in von Hippel-Lindau disease and suggests molecular mechanisms of tumorigenesis. Proteins. 2009; 77: 84–96. doi: 10.1002/prot.22419 19408298

[25] Hon WC, Wilson MI, Harlos K, Claridge TD, Schofield CJ, Pugh CW, et al. Structural basis for the recognition of hydroxyproline in HIF-1 alpha by pVHL. Nature. 2002; 417: 975–978. doi: 10.1038/nature00767 12050673

[26] Min JH, Yang H, Ivan M, Gertler F, Kaelin WG, Jr., Pavletich NP. Structure of an HIF-1alpha -pVHL complex: hydroxyproline recognition in signaling. Science. 2002; 296: 1886–1889. doi: 10.1126/science.1073440 12004076

[27] Hwang S, Ku CR, Lee JI, Hur KY, Lee MS, Lee CH, et al. Germline mutation of Glu70Lys is highly frequent in Korean patients with von Hippel-Lindau (VHL) disease. J Hum Genet. 2014; 59: 488–493. doi: 10.1038/jhg.2014.61 25078357

[28] Miller F, Kentsis A, Osman R, Pan ZQ. Inactivation of VHL by tumorigenic mutations that disrupt dynamic coupling of the pVHL.hypoxia-inducible transcription factor-1alpha complex. J Biol Chem. 2005; 280: 7985–7996. doi: 10.1074/jbc.M413160200 15611064

[29] Lee JS, Lee JH, Lee KE, Kim JH, Hong JM, Ra EK, et al. Genotype-phenotype analysis of von Hippel-Lindau syndrome in Korean families: HIF-α binding site missense mutations elevate age-specific risk for CNS hemangioblastoma. BMC Med Genet. 2016; 17: 48. doi: 10.1186/s12881-016-0306-2 27439424 PMC4955248

