# Supplementary material for: Genotype-phenotype correlation of ocular von Hippel-Lindau disease in Koreans
Source: PLoS One. 2024 Oct 7;19(10):e0311665. doi: 10.1371/journal.pone.0311665 (PMC11458008; doi:10.1371/journal.pone.0311665)

**S1 Figure. Representative fundus photographs and angiographs for each VHL mutation group: (A) Truncating Variant Group (TR), (B) Hypoxia-Inducible Factor-1alpha Binding Site Missense Mutations Group (HM), and (C) Non-Hypoxia-Inducible Factor-1alpha Binding Site Missense Mutations Group (nHM).** (A) A 45-year-old female with a heterozygous exon 2-3 deletion in the VHL gene, showing a large retinal hemangioblastoma with extensive exudation in the temporal periphery of the left eye. (B) A 46-year-old male with a heterozygous c.208G>A variant in the VHL gene, who is blind in the left eye due to retinal hemangioblastomas and has multiple retinal hemangioblastomas and traction membrane in the right eye. (C) A 55-year-old male with a heterozygous c.499C>T variant in the VHL gene, showing no retinal hemangioblastomas.


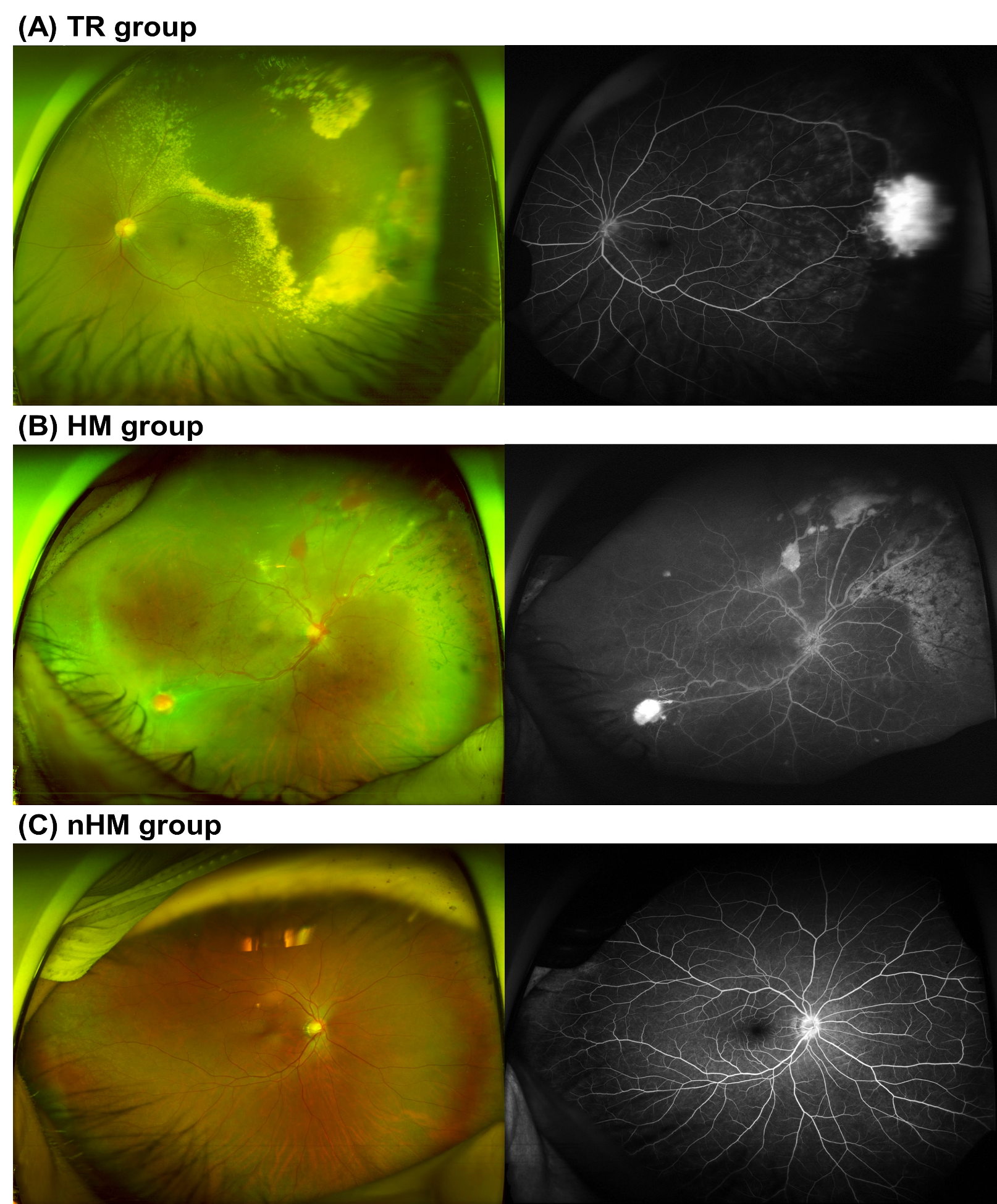

Supplement: S1 Fig — Representative fundus photographs and angiographs for each VHL mutation group: (A) Truncating Variant Group (TR), (B) Hypoxia-Inducible Factor-1alpha Binding Site Missense Mutations Group (HM), and (C) Non-Hypoxia-Inducible Factor-1alpha Binding Site Missense Mutations Group (nHM). (A) A 45-year-old female with a heterozygous exon 2–3 deletion in the VHL gene, showing a large retinal hemangioblastoma with extensive exudation in the temporal periphery of the left eye. (B) A 46-year-old male with a heterozygous c.208G>A variant in the VHL gene, who is blind in the left eye due to retinal hemangioblastomas and has multiple retinal hemangioblastomas and traction membrane in the right eye. (C) A 55-year-old male with a heterozygous c.499C>T variant in the VHL gene, showing no retinal hemangioblastomas. (DOCX) [file pone.0311665.s001.docx]
